# Deficiency of Nox2 prevents angiotensin II-induced inward remodeling in cerebral arterioles

**DOI:** 10.3389/fphys.2013.00133

**Published:** 2013-06-26

**Authors:** Siu-Lung Chan, Gary L. Baumbach

**Affiliations:** Department of Pathology, University of Iowa Carver College of MedicineIowa City, IA, USA

**Keywords:** angiotensin II, hypertrophy, inward remodeling, NADPH oxidase, Nox2, superoxide

## Abstract

Angiotensin II is an important determinant of inward remodeling in cerebral arterioles. Many of the vascular effects of angiotensin II are mediated by reactive oxygen species (ROS) generated from homologs of NADPH oxidase with Nox2 predominating in small arteries and arterioles. Therefore, we tested the hypothesis that superoxide generated by Nox2 plays a role in angiotensin II-induced cerebral arteriolar remodeling. We examined Nox2-deficient and wild-type (WT) mice in which a pressor or a non-pressor dose of angiotensin II (1000 or 200 ng/kg/day) or saline was infused for 4 weeks via osmotic minipumps. Systolic arterial pressure was measured by a tail-cuff method. Pressure and diameter of cerebral arterioles were measured through an open cranial window in anesthetized mice. Cross-sectional area (by histology) and superoxide level (by hydroethidine staining) of cerebral arterioles were determined *ex vivo*. The pressor, but not the non-pressor, dose of angiotensin II significantly increased systolic arterial pressure in both WT and Nox2-deficient mice. Both doses of angiotensin II increased superoxide levels and significantly reduced external diameter in maximally dilated cerebral arterioles in WT mice. Increased superoxide and inward remodeling were prevented in Nox2-deficient mice. Moreover, only the pressor dose of AngII increased cross-sectional area of arteriolar wall in WT mice and was prevented in Nox2-deficient mice. In conclusion, superoxide derived from Nox2-containing NADPH oxidase plays an important role in angiotensin II-mediated inward remodeling in cerebral arterioles. This effect appears to be independent of pressure and different from that of hypertrophy.

## Introduction

Stroke is a common cause of death and disability in developed countries and it is estimated that about 800,000 people have stroke each year in the United States (Goldstein et al., 2011). Chronic hypertension, an important risk factor of stroke, causes profound changes in cerebral vascular structure, such as hypertrophy and inward remodeling (Heagerty et al., 1993). To distinguish it from hypertrophy, we define inward remodeling as a reduction in external diameter due to rearrangement of existing components around a smaller lumen that cannot be accounted for by a reduction in vessel distensibility (Baumbach and Heistad, 1989). It is generally assumed that hypertrophy and inward remodeling play a central role in production of, and protection against, stroke during chronic hypertension (Izzard et al., 2005).

The renin-angiotensin system plays an important role in regulating cerebral vascular structure. Angiotensin II (AngII) is one of several determinants, along with other humoral factors, sympathetic nerves and increases in arterial pressure *per se*, that may result in hypertrophy of cerebral arterioles (Baumbach et al., 1989, 1991, 2003). In contrast, determinants of cerebral arteriolar inward remodeling appear to be limited primarily to AngII (Chillon and Baumbach, 1999; Didion and Faraci, 2003). Unlike hypertrophy, the molecular mechanisms activated by AngII that ultimately lead to inward remodeling remain largely unknown.

Growing evidence suggests that reactive oxygen species (ROS) generated by the renin-angiotensin system may act as second messengers in pathways that lead to vascular changes. Superoxide scavengers or inhibitors of NADPH oxidase prevent AngII-induced endothelial dysfunction in the cerebral circulation (Didion and Faraci, 2003). Moreover, increases in ROS due to a deficiency of superoxide dismutase have been shown to cause hypertrophy, but not inward remodeling, in cerebral arterioles (Baumbach et al., 2006). We are unaware of any previous study that directly addresses the role of ROS in the production of AngII-induced inward remodeling and hypertrophy in the cerebral circulation.

AngII stimulates production of superoxide through one or more of the several known homologs of NADPH oxidase (Cai et al., 2003). The expression and activity of the NADPH oxidase homolog, Nox2, is higher in cerebral arteries than in systemic arteries (Miller et al., 2009). In addition, Nox2 is the predominant NADPH oxidase in smooth muscle derived from small resistance arteries (Touyz et al., 2002), and AngII-induced impairment of endothelial function and cerebral blood flow is prevented in Nox2-deficient (−/−) mice (Girouard et al., 2006; Chrissobolis et al., 2012). These findings suggest that Nox2 may be involved in AngII-mediated changes in the cerebral circulation. Therefore, the aim of this study was to test the hypothesis that deficiency of Nox2 prevents AngII-induced inward remodeling and hypertrophy in cerebral arterioles. To control for the effects of hypertension on cerebral arteriolar structure, we examined effects of a non-pressor, as well as a pressor, dose of AngII.

## Materials and methods

All protocols and procedures conform to the National Institutes of Health Guide for the Care and Use of Laboratory Animals and were approved by the Institutional Animal Care and Use Committee of the University of Iowa.

### Animals

Studies were conducted in 3-month-old male and female Nox2−/− mice (*n* = 30) and wild-type (WT) littermates (*n* = 30). Male-to-female ratio in each experiment and animal group was 50%. Nox2−/− mice and WT littermates were derived from heterozygous Nox2−/− mice (Jackson Laboratories, Bar Harbor, ME, USA). Compared to age-matched WT littermates, the Nox2−/− strain of mice from the Jackson Laboratories have similar body weights and normal function of heart and kidneys. Breeding and genotyping were performed in a virus- and pathogen-free barrier facility at the University of Iowa.

### Angiotensin II treatment and measurement of conscious blood pressure

Osmotic minipumps (Alzet, model 1004, Durect, Cupertino, CA, USA) were implanted subcutaneously in the midscapular region in mice during anesthesia with ketamine/xylazine (87.5/12.5 mg/mL, 10 ml/kg, i.p.). The minipumps were used to continuously infuse vehicle (isotonic saline) or AngII (1000 or 200 ng/kg/min) for a period of 28 days.

Systolic blood pressure (BP) was measured using an automated tail-cuff device (BP-2000, Visitech Systems). Prior to implantation of minipumps, mice were trained for 5 days and baseline BP was recorded, followed by minipump implantation and measurements of BP on day 7, 14, 21, and 28 of AngII treatment. Each day thirty BP measurements were made for each mouse and data were averaged.

### Measurement of cerebral arteriolar pressure and diameter

Animals were weighed and anesthetized with ketamine/xylazine (87.5/12.5 mg/ml, 10 ml/kg, i.p.). Supplementary anesthetic (pentobarbital sodium, 50 mg/ml) was given during the experiment through a catheter connected to the femoral vein. Systemic systolic, diastolic, mean, and pulse pressure were measured continuously via catheter connected to femoral arteries. The depth of anesthesia was assessed every 15 min by toe pinch. Pressure and internal diameter were measured in first-order cerebral arterioles on the cerebrum through an open cranial window *in vivo* (Baumbach et al., 2003). Cerebral arteriolar systolic, diastolic, mean, and pulse pressure were measured continuously with a glass micropipette connected to a servo-null pressure-measuring device (model 5, Instrumentation for Physiology and Medicine, Inc.). Approximately 30 min after completion of surgery, measurements of cerebral arterioles were obtained under baseline conditions. To evaluate passive characteristics of cerebral arterioles, vascular muscle was deactivated by suffusion of cerebral vessels with artificial cerebrospinal fluid containing EDTA (67 mmol/L), which produces maximal dilatation of cerebral arterioles (Baumbach et al., 1988). Pressure-internal diameter relationships were obtained in maximally dilated arterioles between arteriolar mean pressures of 40 and 10 mmHg by stepwise controlled hemorrhage through a catheter connected to the femoral vein. Internal diameter of arterioles was continuously recorded through a microscope connected to a closed-circuit video system with a final magnification of ×356. Internal diameter was measured from digitized images of arterioles using Image J (National Institute of Health, Bethesda, MD, USA).

### Measurement of cerebral arteriolar cross-sectional area

To evaluate cross-sectional area (CSA) of the arteriolar wall, arterioles were fixed at physiological pressure *in vivo* by suffusion of vessels with fixative (2.25% glutaraldehyde in 0.10 mol/L cacodylate buffer) while maintaining cerebral arteriolar pressure at physiological levels the same as before stepwise hemorrhage. After the anesthetized animal was euthanized using overdose sodium pentobarbital, a cerebral arteriolar segment was removed, processed, and embedded in Spurr's low viscosity resin while maintaining cross-sectional orientation. Samples were cut into 1-μm sections, stained with Richerson's stain and CSA was determined histologically by Image J. CSA of the cerebral arteriolar wall was determined by tracing the inner and outer edges of the vessel wall using Image J, which yielded lumen area (CSA_L_) and the area of the vessel wall plus the lumen area (CSA_W+L_). CSA of the vessel wall was calculated by subtracting CSA_L_ from CSA_W+L_. A key advantage of this method is that tissue volume is well preserved (Lee et al., 1980) as compared to fixation with formaldehyde and paraffin embedding (Hart and O'Donnell, 1980), which typically results in a 20–30% shrinkage in tissue volume. Another advantage is that the 1-μm thick plastic embedded sections increase accuracy in CSA measurements by providing a higher resolution of light microscopic detail than sections of paraffin embedded tissue, which are typically 5–6 μm in thickness.

### Measurement of superoxide

Levels of superoxide were evaluated in 6–8 μm thick frozen sections of unfixed cerebral arterioles using hydroethidine-based (2 μmol/L hydroethidine) confocal microscopy as described previously (Didion et al., 2002). Laser settings were identical for acquisition of images, and vessels from WT and Nox2−/− mice were processed and imaged in parallel. Relative increases in ethidium fluorescence were determined and normalized to the CSA of the vessel wall.

### Calculation of mechanical characteristics

The methods that we used to calculate circumferential stress and strain of arterioles on the surface of the cerebrum have been described in detail (Chillon and Baumbach, 2004). Circumferential stress (σ) was calculated from cerebral arteriolar pressure (*P*), internal diameter of cerebral arterioles (*D*_*i*_), and wall thickness (WTh): σ = (*P* × *D*_*i*_)/(2WTh). Cerebral arteriolar pressure was converted from mmHg to newtons/m^2^ (1 mmHg = 1.334 × 10^2^ N/m^2^). WTh was calculated from histological measurements of CSA and *in vivo* measurements of D_*i*_: WTh = [(4CSA/π + *D*^2^_*i*_)^1/2^ – *D*_*i*_]/2. External diameter of cerebral arterioles (*D*_*e*_) is calculated as: *D*_*e*_ = *D*_*i*_ + 2WT. Histological determinations of CSA were used in all calculations of WTh and circumferential stress. Circumferential strain (ε) was calculated as ε = (*D*_*i*_ − *D*_*o*_)/*D*_*o*_, where *D*_*o*_ was original diameter. We defined original diameter as the diameter at 10 mmHg pressure. Elastic modulus (λ) was defined as the slope of the stress-strain curve and calculated as σ/ε.

### Statistical analysis

All data are presented as mean ± SEM. Differences between individual groups were determined by ANOVA with *post-hoc* Bonferroni test, using Graph Pad Prism 5 (Graph Pad Software, Inc., San Diego, CA, USA). Differences were considered significant when *P* < 0.05.

## Results

### Nox2 deficiency does not alter pressor responses of angiotensin II

Pretreatment levels of systolic arterial BP (SBP) were not significantly different in WT and Nox2−/− mice (Table 1). Treatment with saline or the non-pressor dose of AngII had no effect on SBP in either WT or Nox2−/− mice. The pressor dose of AngII, as expected, significantly increased SBP in WT and Nox2−/− mice 7 days after the initiation of treatment. SBP remained significantly elevated in both WT and Nox2−/− mice during the subsequent 3 weeks of treatment with the pressor dose of AngII. Thus, deficiency of Nox2 did not alter the pressor response to AngII.

**Table 1:** **Physiological data**.

**Parameters**	**WT Saline**	**WT AII-1000**	**WT AII-200**	**−/− Saline**	**−/− AII-1000**	**−/− AII-200**
Systolic Arterial						
Pressure (mmHg)						
Day 0	110 ± 4	115 ± 3	109 ± 4	116 ± 4	104 ± 3	107 ± 4
Day 7	106 ± 3	135 ± 5^*^	111 ± 3	110 ± 5	142 ± 8^*^	109 ± 6
Day 14	112 ± 3	134 ± 3^*^	115 ± 4	109 ± 5	139 ± 4^*^	109 ± 5
Day 28	116 ± 4	128 ± 4^*^	112 ± 4	113 ± 4	133 ± 5^*^	112 ± 3
Cerebral Arterioles						
Pressure (mmHg)						
Systolic	49 ± 4	47 ± 3	57 ± 2	48 ± 4	46 ± 4	53 ± 2
Diastolic	34 ± 3	34 ± 2	41 ± 1	34 ± 3	35 ± 3	39 ± 2
Mean	39 ± 3	38 ± 3	46 ± 1	39 ± 3	39 ± 4	44 ± 2
Pulse	15 ± 1	13 ± 1	16 ± 1	14 ± 1	11 ± 1	14 ± 1
Elastic Modulus	6.7 ± 0.7	5.1 ± 0.4^*^	6.9 ± 0.3	6.7 ± 0.8	5.9 ± 0.5	6.3 ± 0.6
Arterial Blood Gases						
pH	7.35 ± 0.04	7.34 ± 0.02	7.38 ± 0.02	7.38 ± 0.03	7.39 ± 0.02	7.35 ± 0.02
pCO_2_	30 ± 4	33 ± 2	28 ± 1	24 ± 2	29 ± 2	32 ± 2
pO_2_	112 ± 7	99 ± 4	107 ± 5	113 ± 9	105 ± 8	94 ± 4
Age (week)	16.6 ± 0.9	17.8 ± 0.8	17.6 ± 1.0	17.4 ± 0.9	18.5 ± 0.8	17.9 ± 0.9
Weight (g)	26.1 ± 1.3	26.4 ± 2.0	26.8 ± 1.3	24.5 ± 1.0	23.8 ± 1.5	25.8 ± 0.9
*n*	10	10	10	10	10	10

Systolic arterial pressure, cerebral arteriolar pressure, internal diameters, elastic modulus, and arterial blood gases in wild-type (WT) and Nox2 deficient (−/−) mice treated with angiotensin II (AII, 200 or 1000 ng/kg/min). Systolic arterial pressure was measured using the tail-cuff method in conscious mice. Cerebral arteriolar pressure was measured under anesthetized condition. Values are mean ± SEM.

* P < 0.05 vs. WT saline group.

### Nox2 deficiency prevents angiotensin II-mediated superoxide production

**Figure 1:**
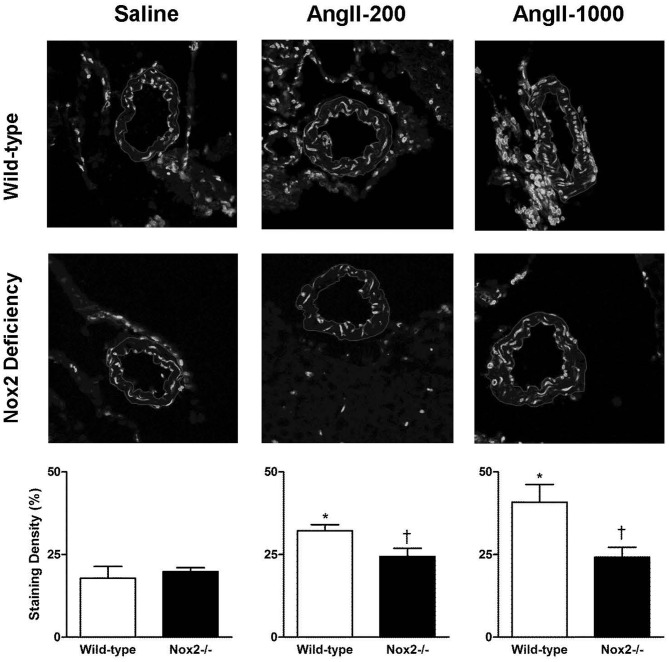
**Representative confocal fluorescent sections (inner and outer edges of cerebral arterioles are highlighted) and relative fluorescence in cerebral arterioles in wild-type (WT) and Nox2-deficient (−/−) mice treated with a pressor (AngII-1000) or a non-pressor (AngII-200) dose of angiotensin II or saline**. Data are presented as mean ± SEM of 5–6 mice. ^*^P < 0.05 vs. saline-treated WT group; ^†^*P* < 0.05 vs. corresponding WT group.

Figure 1 shows representative micrographs and quantification of ethidium fluorescence in cerebral arterioles (Figure 1). Quantification of ethidium signal revealed basal levels of superoxide to be similar in cerebral arterioles of saline-treated Nox2−/− mice and WT mice. Ethidium signal was significantly higher in cerebral arterioles in WT mice treated with both the non-pressor and pressor doses of AngII. In addition, the pressor dose of AngII increased relative fluorescent intensity in WT mice by about 100%, while the non-pressor dose increased intensity by about 50%. In contrast to WT mice, cerebral arteriolar fluorescence was unchanged during treatment of Nox2−/− mice with either dose of AngII relative to Nox2−/− mice treated with saline.

### Nox2 deficiency prevents changes of cerebral arteriolar structure by angiotensin II

**Figure 2:**
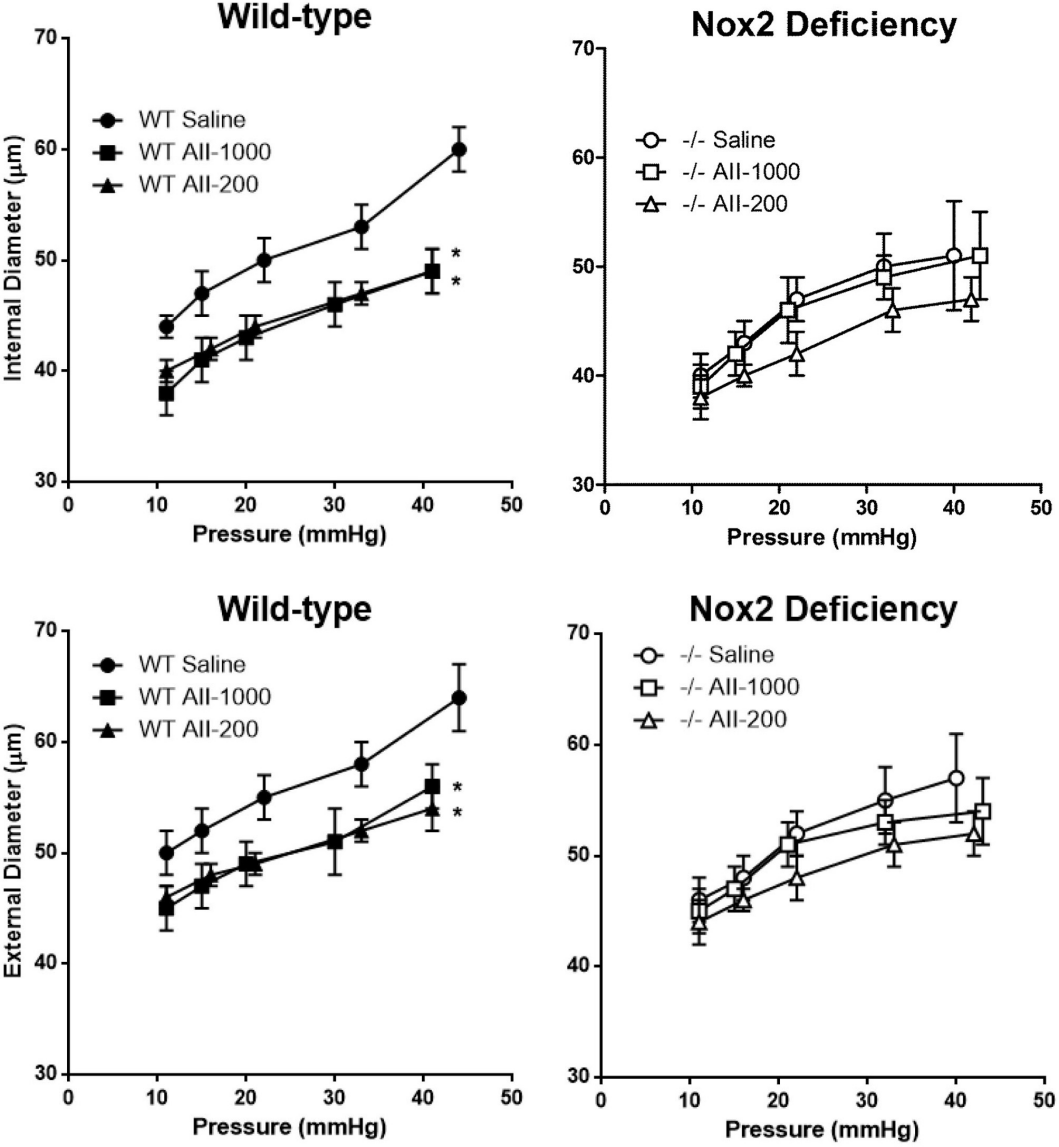
**Pressure-internal and external diameter relationships in maximally dilated cerebral arterioles in wild-type (WT) or Nox2-deficient (−/−) mice treated with a pressor (AII-1000) or a non-pressor (AII-200) dose of angiotensin II or saline**. Data are presented as mean ± SEM of 10 mice. ^*^P < 0.05 vs. saline-treated WT group.

Internal and external diameters in maximally dilated cerebral arterioles were not significantly different in saline-treated WT and Nox2−/− mice. Both internal and external diameters were significantly decreased by the pressor, as well as the non-pressor, dose of AngII at all levels of arteriolar pressure between 10 and 40 mmHg in WT mice (Figure 2). In Nox2−/− mice, on the other hand, internal and external diameters were not significantly different in either of the AngII treated groups than in the saline treated group at any level of arteriolar pressure. Therefore, these results showed that Nox2 deficiency prevented AngII-induced inward remodeling in cerebral arterioles.

**Figure 3:**
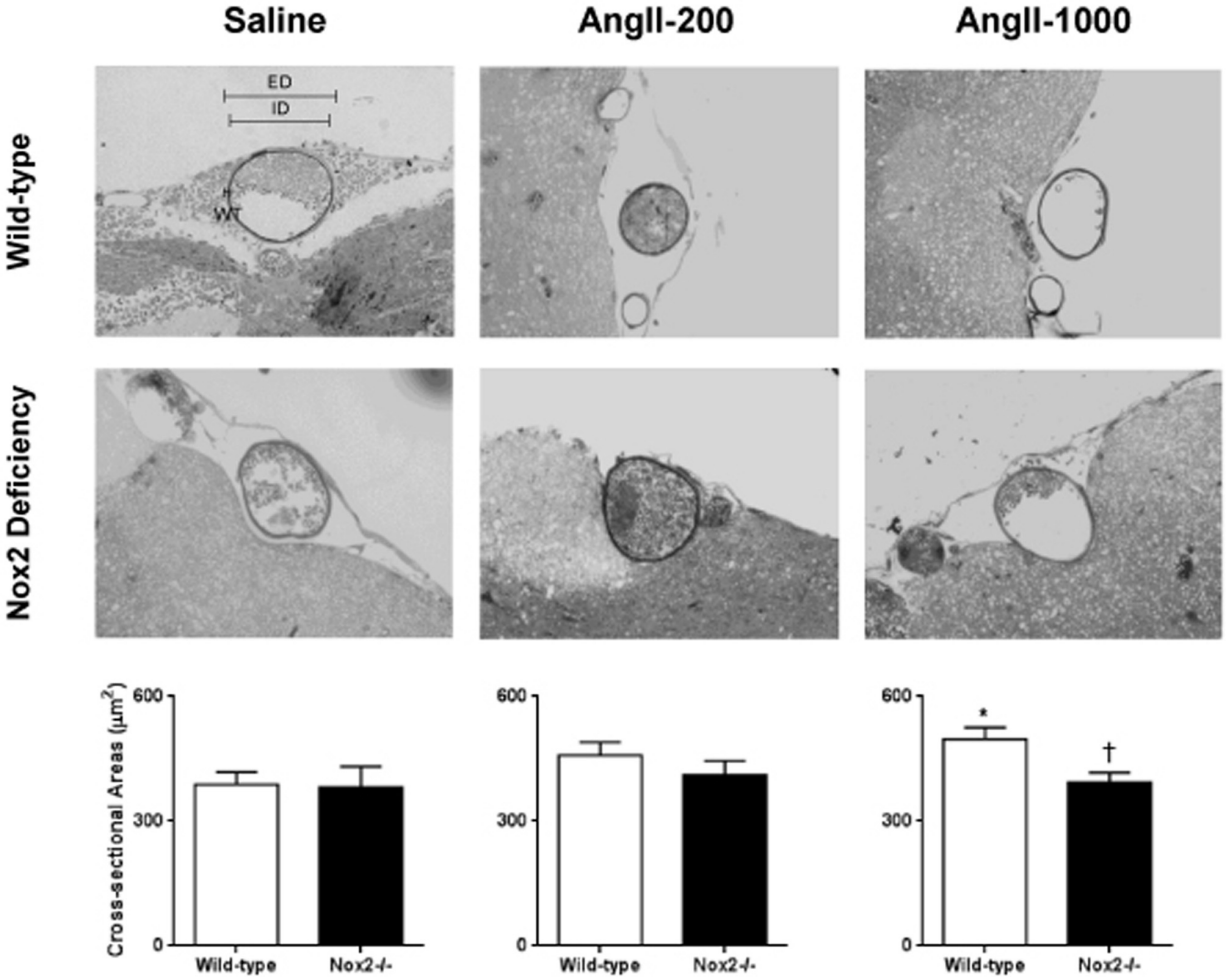
**Representative micrographs of histological sections and cross-sectional area (CSA) of maximally dilated cerebral arterioles in wild-type (WT) and Nox2-deficient (−/−) mice treated with a pressor (AngII-1000) or a non-pressor (AngII-200) dose of angiotensin II or saline**. Data are presented as mean ± SEM of 10 mice. ^*^P < 0.05 vs. saline-treated WT group; ^†^*P* < 0.05 vs. AngII-treated WT group. ED, external diameter; ID, internal diameters; WT, wall thickness.

CSA of the cerebral arteriolar wall was significantly increased in pressor, but not the non-pressor, dose of AngII in WT mice (Figure 3). In contrast, neither dose of AngII significantly altered CSA of the arteriolar wall in Nox2−/− mice. This finding indicates that deficiency of Nox2 prevented AngII-induced hypertrophy of cerebral arterioles.

**Figure 4:**
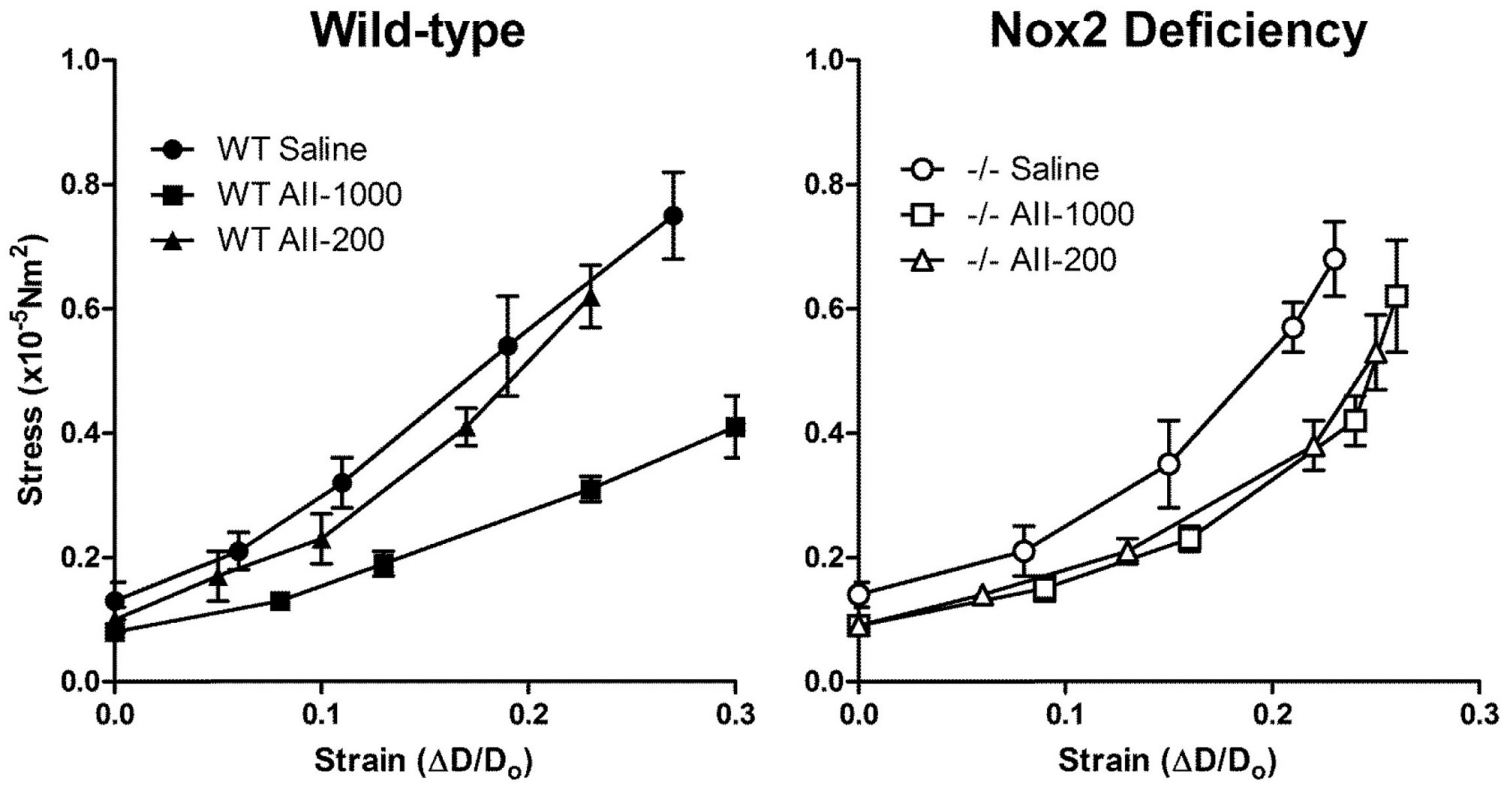
**Stress-strain relationships in maximally dilated cerebral arterioles in wild-type (WT) and Nox2-deficient (−/−) mice treated with a pressor (AII-1000) or a non-pressor (AII-200) dose of angiotensin II or saline**. Data are presented as mean ± SEM of 10 mice.

The stress-strain curve in cerebral arterioles treated with the pressor, but not the non-pressor, dose of AngII was shifted to the right in arterioles treated with saline in WT mice (Figure 4). The slope of tangential elastic modulus vs. stress was decreased in cerebral arterioles in the pressor AngII group relative to the non-pressor AngII and saline group (Table 1). In Nox2−/− mice, the stress-strain curves in cerebral arterioles treated with both the pressor and non-pressor doses of AngII were shifted to the right of the curve in arterioles treated with saline, but to a lesser degree than observed in WT mice (Figure 4). The slope of tangential elastic modulus vs. stress was not significantly different in either the pressor or non-pressor group relative to the saline group (Table 1). These findings suggest that deficiency of Nox2 may attenuate AngII-induced increases in distensibility of cerebral arterioles.

## Discussion

There are several major new findings in this study. First, both the non-pressor and pressor doses of AngII increased superoxide levels in cerebral arterioles in WT, but not in Nox2−/− mice. Increases in cerebral arteriolar superoxide in WT mice were greater with the pressor dose of AngII than with the non-pressor dose. Second, the non-pressor dose of AngII caused inward remodeling of cerebral arterioles in WT mice that was prevented in Nox2−/− mice. These results suggest that Nox2-derived superoxide, independent of increase in pressure, has an important role in AngII-mediated inward remodeling. Third, the mechanism of AngII-mediated inward remodeling of cerebral arterioles appears to be different from that of hypertrophy. Only the pressor dose of AngII resulted in hypertrophy of cerebral arterioles, suggesting pressor effect may be mandatory for hypertrophy. Together, we showed that AngII-induced superoxide production from Nox2-containing NADPH oxidase has important role in inward remodeling, which is mechanistically different from that of hypertrophy.

AngII stimulates production of ROS in the vessel wall via activation of membrane-bound NADPH oxidases. The distribution of NADPH oxidase homologs Nox1, Nox2, and Nox4 appears to be dependent on cell type and vessel size. Nox1 is the predominant NADPH oxidase homolog in smooth muscle derived from large arteries (Lassegue et al., 2001), whereas Nox2 predominates in smooth muscle derived from small resistance arteries (Touyz et al., 2002). In this study, we used ethidium fluorescence to show that AngII stimulates superoxide production in cerebral arterioles through activation of Nox2. This finding supports the concept that Nox2 is the predominant source of superoxide in arterioles in the cerebral circulation. We are aware of the potential problems inherent in using ethidium staining as an indicator of superoxide production, such as the introduction of non-specific fluorescent products. To address these concerns, we kept all conditions of staining and settings of confocal microscopy consistent for all samples and compared staining density between groups semi-quantitatively.

Production of ROS derived from NADPH oxidase plays an important role in AngII-induced hypertension (Ortiz et al., 2001). It is clear that Nox1 enhances pressor effects of AngII (Dikalova et al., 2005; Gavazzi et al., 2006). On the other hand, the contribution of Nox2-generated superoxide to AngII-induced increases in BP remains debatable. Previous studies found that Nox2 deficiency either diminishes (Wang et al., 2001) or has no effect (Touyz et al., 2005) on the pressor response to AngII. Our finding in this study supports the concept that Nox2 has no significant effect on AngII-induced hypertension. Moreover, the non-pressor dose of AngII did not increase BP, although a significant increase in superoxide was detected. In contrast to our finding, the same dose of AngII that did not produce a pressor response in this study has been reported to slightly increase BP (about 10%) after 3 weeks in at least two previous studies (Kawada et al., 2002; Izumiya et al., 2003). We speculate the difference may be due to different mouse colonies from different animal providers. For example, inbred C57/B6 mice from Taconic Laboratories (Germantown, NY, USA) were used in a previous study (Kawada et al., 2002) vs. inbred Nox2 mice that have C57/BL6J background from Jackson Laboratories.

Inward remodeling of small arteries and arterioles is thought to be an adaptive response to hypertension that normalizes wall stress and thus protects against the damaging effects of elevated pressure (Izzard et al., 2005). The renin-angiotensin system has been identified as an important determinant of inward remodeling, as evidenced by the findings that treatment with an angiotensin-converting enzyme inhibitor attenuates cerebral arteriolar inward remodeling (Chillon and Baumbach, 1999), and overexpression of human renin and angiotensinogen results in inward remodeling of cerebral arterioles in mice (Baumbach et al., 2003). Furthermore, the importance of the renin-angiotensin system to vascular inward remodeling has been confirmed in human essential hypertension (Thybo et al., 1995). Our findings in this study provide additional confirmation of the important role of AngII in inward remodeling of cerebral arterioles.

Superoxide derived from NADPH oxidase has been shown to mediate many of the effects induced by AngII (Griendling et al., 1994; Rajagopalan et al., 1996; Ushio-Fukai et al., 1999). A major goal of this study was to examine the role of ROS in AngII-induced remodeling. Our finding that deficiency of Nox2 prevented AngII-induced inward remodeling in cerebral arterioles suggests that ROS derived from Nox2-containing NADPH oxidase may act as an important mediator in inward remodeling. In addition, this result corroborates the finding in a previous study showing that ROS is involved in inward remodeling of systemic resistance arteries (Martinez-Lemus et al., 2011). We speculate that ROS may promote AngII-induced inward remodeling of cerebral arterioles through mechanisms that modulate migration of vascular smooth muscle (VSM). This possibility is based on the observations that AngII is an important promoter of VSM migration (Bell and Madri, 1990), and ROS play an important role in several signaling domains involved in cellular migration (San Martin and Griendling, 2010). As an example, nitric oxide (NO) has been found to inhibit AngII-induced migration of VSM *in vitro* (Dubey et al., 1995), which suggests the possibility that generation of superoxide by AngII may lead to inward remodeling by destroying NO thereby removing its inhibitory influence on VSM migration.

Another goal in the present study was to separate pressor and non-pressor effects of AngII on cerebral arteriolar remodeling. The possibility that AngII might contribute directly to inward remodeling independently of its pressor effects was suggested by our previous findings that (1) a low dose of an angiotensin converting enzyme inhibitor, perindopril, was nearly as effective as the high dose in attenuating inward remodeling of cerebral arterioles in stroke-prone spontaneous hypertensive rats, even though the low dose was half as effective as the high dose in lowering cerebral arteriolar pressure, and (2) in contrast to the low dose of perindopril, the β-blocker, propranolol, did not significantly attenuate inward remodeling of cerebral arterioles in stroke-prone spontaneous hypertensive rats, even though it was much more effective than the low dose of perindopril in lowering cerebral arteriolar pressure (Chillon and Baumbach, 1999). Our finding in this study that the non-pressor dose of AngII resulted in the same degree of inward remodeling in cerebral arterioles as the pressor dose provides strong support for the hypothesis that AngII induces inward remodeling directly, and independently, of its pressor effect.

The mechanism responsible for AngII-induced hypertrophy of cerebral arterioles appears to differ from that of AngII-induced inward remodeling, as suggested by the finding that the non-pressor dose of AngII did not result in cerebral arteriolar hypertrophy, but was sufficient to induce inward remodeling. This finding suggests that in contrast to inward remodeling, increased pressure, rather than AngII *per se*, is an important determinant of cerebral arteriolar hypertrophy. This concept is arguable, however, because suppression of the pressor effect of AngII may or may not prevent vascular hypertrophy (Griffin et al., 1991; Chillon and Baumbach, 1999). In addition, AngII-induced hypertrophy is ROS-dependent, as suggested by the result that deficiency of Nox2 prevented hypertrophy of cerebral arterioles in response to AngII. This result also supports the concept that Nox2 is the predominant NADPH oxidase responsible for producing ROS and plays an important role in Ang II-induced hypertrophy of cerebral arterioles.

In conclusion, we showed for the first time that superoxide derived from Nox2-containing NADPH oxidase has critical role in inward remodeling of cerebral arterioles mediated by AngII. This effect is independent of increases in pressure and appears to differ from that of hypertrophy.

### Conflict of interest statement

The authors declare that the research was conducted in the absence of any commercial or financial relationships that could be construed as a potential conflict of interest.
